# Femoral alloprosthesis in bone defect of 30 cm as extremity salvage

**DOI:** 10.1016/j.tcr.2024.101082

**Published:** 2024-07-29

**Authors:** Diego de Francisco Jiménez Cortes, Edgar Manuel Bodu Lamberti

**Affiliations:** Palermo Clinic, Calle 45c #22-02, Bogotá, Colombia

**Keywords:** Osteomyelitis, Prosthesis implantation, Allografts, Case report (MeSH)

## Abstract

Defects in femoral bone segments represent a reconstructive challenge; they are caused secondary to multiple and extensive debridement in cases of patients with infections, tumors or high-energy trauma. Different treatments have been proposed to address this problem, however, these are limited when it comes to large defects that generate instability of the implants in the native bone as well as loss of functionality and length of the extremities. In the proximal femur, allograft prosthesis composites have been described in the management of extensive tumor resections, but they are not yet widely used in the management of bone defects due to osteomyelitis. The case of a 51-year-old male patient with post-traumatic pan-osteomyelitis of the femur Cierny-Mader IV with a 30-centimeter defect in whom limb salvage was achieved through the application of a femoral alloprosthesis is presented, exhibiting this surgical technique as an alternative in ample resections secondary to infectious processes in young patients, furthermore, offering a solution to the shortage of some prosthetic components in our surrounding.

## Introduction

Osteomyelitis is a complex pathology that involves progressive bone destruction and often represents a therapeutic challenge for the orthopedic surgeon. Multiple etiologies have been described, most frequently being hematogenous dissemination or as a sequela of the management of high-energy trauma, reporting an incidence of 9–55 % in open fractures Gustilo and Anderson III [1]. Its pathogenesis is mainly characterized by chronic pyogenic bone inflammation, triggering bone devascularization and necrosis. Antibiotic therapy is often not enough to achieve adequate infectious control due to insufficient vascular supply of the adjacent tissues. Considering the above, multiple strategies and therapeutic approaches have been developed, but in the end all of them will lead to the loss of bone stock 2.

In 1852, the French surgeon Edouard Chassaignac described osteomyelitis for the first time, emphasizing early debridement and amputation as the main therapeutic strategy. At present, aggressive debridement continues to be the basis and pillar of treatment, aiming at the resection of devitalized bone until a punctate haversian bleeding (paprika sign) is achieved [3]. In addition, targeted antibiotic therapy and the identification of the specific anatomical location means that almost all of these patients require a personalized and unique therapeutic effort that leads to the combination and application of multiple surgical techniques for the salvage of a limb [4]. Even so, a high incidence of lower limb amputations is reported, ranging from 7.2 % in patients without comorbidities to 75 % in those with diabetes mellitus, heart failure and peripheral arterial disease [5].

Moreover, it is associated with a high recurrence of symptoms and fistulous tracts in 20–30 % [6], which implies an increase in morbimortality and in the economic burden of the health system. We report the case of a patient with post-traumatic pan-osteomyelitis of the left femur Cierny-Mader IV and a segmental bone defect in whom a personalized management was performed with the implementation of several surgical techniques as a limb salvage strategy.

## Case presentation

**Fig. 1:**
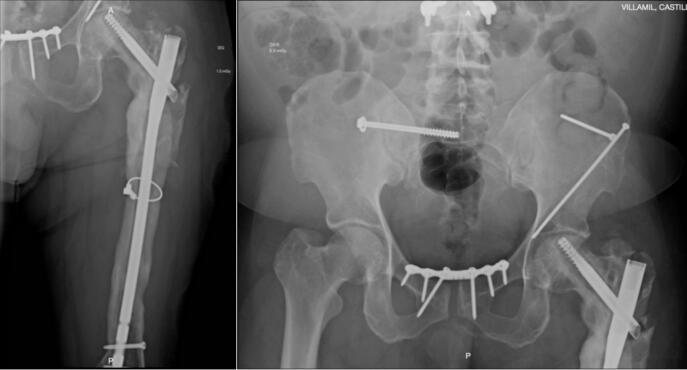
Consolidation of pelvic ring fracture with symphysis plating and sacroiliac arthrodesis, left proximal femur with loss of cortical congruence and bone sequestrations extending throughout the entire metaphyseal bone.

A 51-year-old male patient, with a history of a fall from a height of more than 20 m during sporting activity (paragliding), requiring multiple surgical interventions in different health institutions in Bogotá, Colombia, to whom hip disarticulation was proposed as part of the management of his pathology. He was admitted to the emergency department with pain in the left lower limb associated with multiple functional fistulas in the thigh. Complete blood count, C-Reactive Protein (CRP), and Erythrocyte Sedimentation Rate (ESR) were consistent with an active infectious process and basic radiology studies showed alteration in bone morphology with diffuse thickening of the cortices involving the diaphysis and supracondylar region of the femur (Fig. 1).

**Fig. 2:**
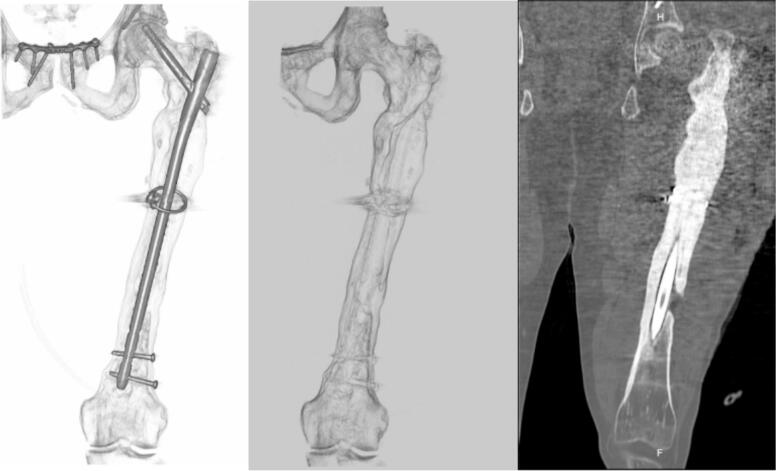
CAT scan with 3D reconstruction showing thickening and circumferential irregularity of the proximal two thirds of the left femur.

The patient was considered to have chronic post-traumatic osteomyelitis of the femur Cierny-Mader IV, and surgery was proposed to perform an initial objective evaluation of the condition and involvement of the limb. He was taken to surgery where multiple fistulous tracts from bone to skin were evidenced, as well as a femoral cortical defect of 11 cm that compromised up to five cm of the upper edge of the femoral condyles. Cultures were taken at different surgical times: five cultures at the beginning of the debridement and five cultures at the end of the procedure. Finally, a subatmospheric pressure system was placed (VAC®). Cultures reported isolation of *Enterococcus faecium*, for which he received antibiotic management by the infectious diseases department with ampicillin. The patient required multiple additional surgical washings, evaluating bone and integumentary defects, as well as clinical and paraclinical control of the infectious process. Once the exacerbation of osteomyelitis was controlled, it was decided to initiate a two-stage limb salvage strategy and a computerized axial tomography (CAT) scan of the left hip and femur with three-dimensional (3D) reconstruction was requested for surgical planning (Fig. 2).

**Fig. 3:**
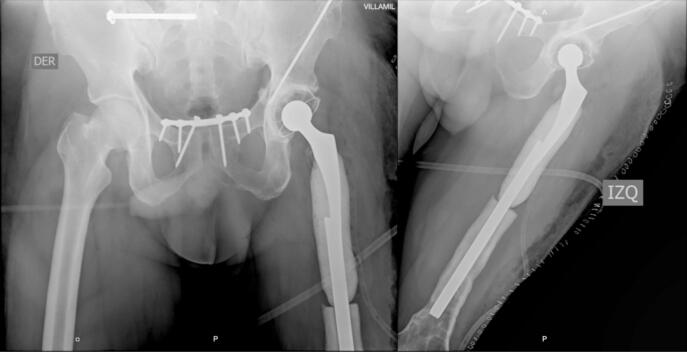
Left hip joint spacer coated with cement and antibiotic custom made using a proximal module of 12/14 × 150 mm with tapper impacted on a 14 × 42 mm Kuntscher nail, 28 × 50 mm cemented cup and a 28 mm femoral head.

In the first surgical stage, removal of the osteosynthesis material and an “en bloc” resection of the necrotic segment of the proximal femur with subsequent elaboration of an articulated cement spacer made-to-measure intraoperatively was performed (Fig. 3). A subatmospheric pressure system and an adjacent drainage system (Hemovac®) were complemented for skin care due to the large defect and for dead space management, avoiding collections in the resected area, which was removed five days later showing scarce drainage. The patient continued antibiotic treatment until completing a total of 42 days.

**Fig. 4:**
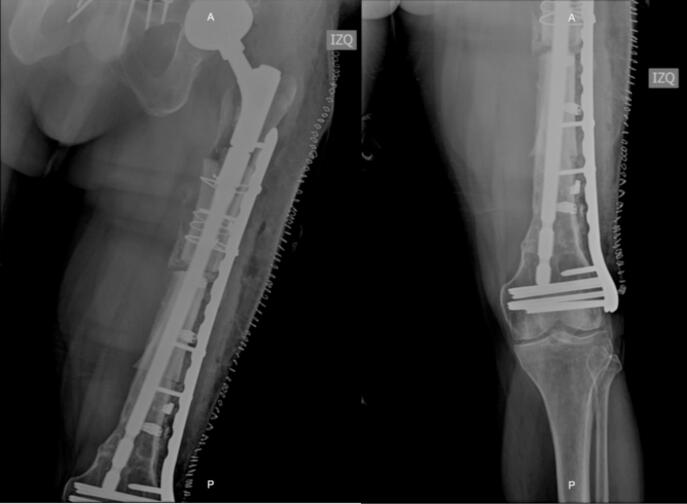
Femur alloprosthesis stabilized by cable systems, periprosthetic wires and condylar support plate.

During seven months, an outpatient follow-up was performed, periodically evaluating CRP and ESR showing no elevation and consequent stabilization of their values until negativization, along with adequate coverage of soft tissues and skin condition. At nine months, the definitive procedure was performed, evidencing intraoperatively a femoral segmental and supracondylar defect of 20 and 10 cm respectively, for a total of 30 cm of bone defect. After surgical planning and provided with the proximal femoral allograft and prosthetic materials (Wagner revision stem), acetabular cup revision was done with a dual mobility cup and then the distal femoral segment was prepared until an adequate press fit of the stem was obtained. Afterwards, by means of trial components, preparation of the femoral allograft was performed; during the intraoperative evaluation and despite the stability of the alloprosthetic complex, augmentation with a condylar support plate was considered for stability of the distal femur (Fig. 4).

**Fig. 5:**
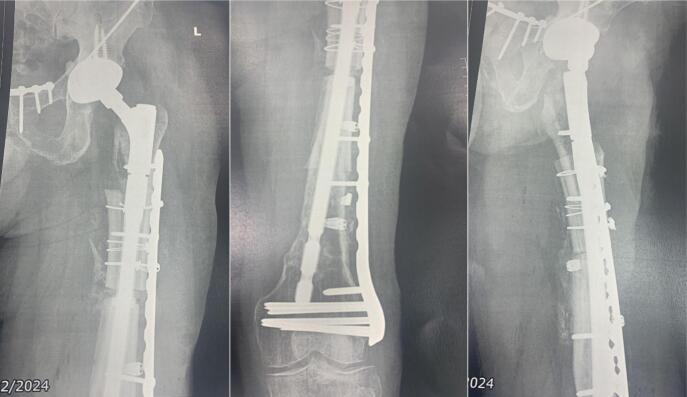
AP and lateral radiograph of the left femur three months post-op showing integration and stabilization of the alloprosthesis with signs of graft resorption at the proximal femur.

**Fig. 6:**
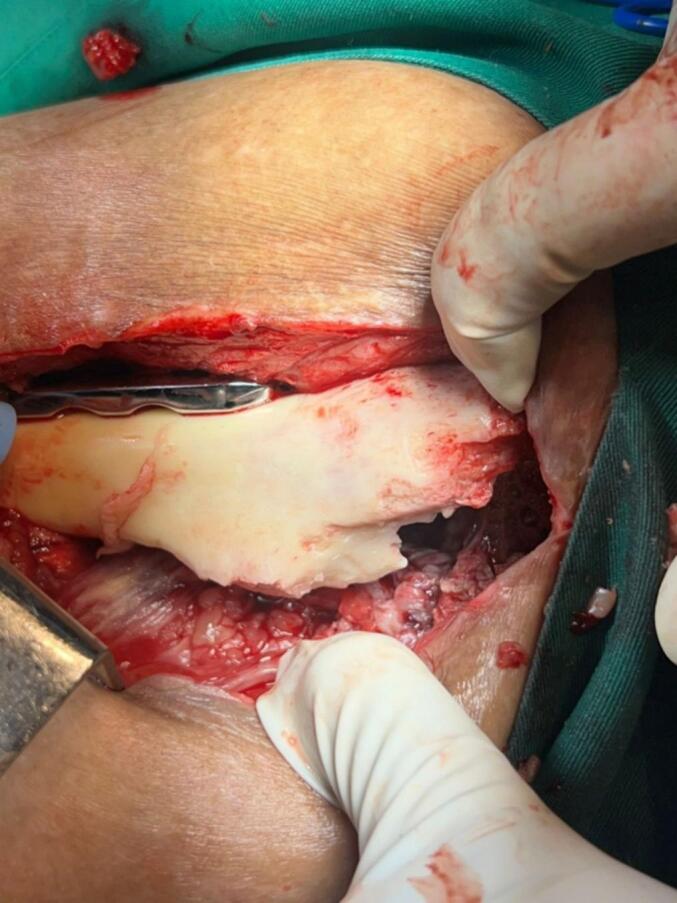
Graft resorption in the anterior region of the proximal femur.

**Fig. 7:**
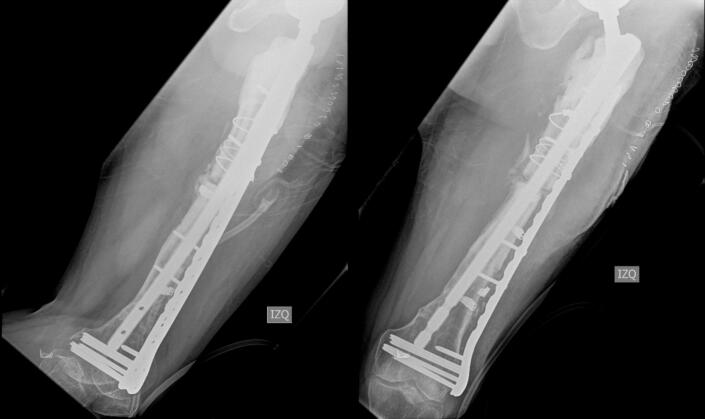
Postoperative AP and lateral radiographs of the femur showing integration and stability of the distal alloprosthetic complex.

The patient remained hospitalized for pain management and postoperative surveillance for one week and was subsequently discharged. Radiographic control at three months post-op showed integration and stabilization of the alloprosthesis with subtle signs of graft resorption at the proximal femur (Fig. 5). The patient was tolerating full weight bearing and mobilizing the extremity satisfactorily. During outpatient follow-up, a secreting fistulous tract in the lateral aspect of the thigh was evidenced in addition to further graft resorption. For this reason, the patient underwent fistulectomy and surgical washing at eight months post-op with intraoperative evidence of approximately 20 % graft resorption in the anterior region of the proximal femur without compromising the stability of alloprosthetic complex in its distal counterpart (Fig. 6, Fig. 7). Cement with antibiotic was used to coat the area of resorption and cultures were taken which reported negative after 72 h of incubation. The patient remained hospitalized for postoperative surveillance and was later discharged without complications.

## Discussion

Allograft prosthesis composites (APC) are an alternative for young patients who have the osteogenic capacity to achieve union between the allograft and native bone. This technique offers the possibility to achieve an adequate coverage of periarticular tissues [7] and therefore allows reestablishment of the abductor lever minimizing the biomechanical risks of an endoprosthesis. However, extensive APC have a higher probability of graft resorption, infection, periprosthetic fracture and non-union [8]. Dubory and colleagues evaluated the clinical and radiological outcomes of a group of 46 patients with femoral APC, reporting a revision-free rate of 54.1 % and graft union of 84.1 % at 10 years, demonstrating graft resorption as the main complication, without affecting functionality or survival of the implant [9].

Gautam and colleagues carried out a meta-analysis of 17 retrospective reviews that studied the functionality between patients with endoprosthesis versus APC, observing better outcomes in the Musculoskeletal Tumor Society Score, favoring the use of APC in both lower and upper limb attributable to the advantage of performing a reinsertion of adjacent tissues [10], as opposed to endoprosthesis that lack an adequate interface for reinsertion [11]. Farid and colleagues evaluated functionality and implant survival in patients with endoprosthesis and APC, observing higher abductor muscle strength and implant survival rates at 5, 10 and 15 years in the APC group [12]. This composition of allograft and prosthesis is being taken up again for the management of bone defects in young patients as it adapts satisfactorily to the high physical demands that characterize this age group.

Currently, its use is supported in Paprosky IV femoral segmental defects, extensive tumor resections and Vancouver B3 periprosthetic fractures, displaying promising results [[13], [14], [15]]. Regarding the management of osteomyelitis, this procedure is rarely applied for the treatment of bone defects resulting from aggressive debridement [16,17]. Still, the patient presented a 30 cm defect extending to the supracondylar region of the femur, hence it was decided to use a femoral APC in search of limb salvage, without closing the possibility of other procedures that the patient may require in the future for the preservation of his limb and motor function [18]. Therefore, the implementation of this surgical technique deserves special attention and continuous improvement; further studies are needed to generate standardization protocols to identify those patients who would benefit from femoral APC, as reported in this case.

## Conclusion

APCs are still in use today; they have already been evaluated in case series and reports in the medical literature, observing diverse results regarding their functionality and complications. We consider that in these young patients, the use of an allograft is another tool that prolongs the life of a limb for a period of more or less 10 years. This gain of time is very useful and does not close the possibility of performing other types of reconstructive procedures in the future with the application of different technologies for limb salvage, such as endoprosthesis or total femur replacement, which are interventions of great value in patients with large bone defects. However, such cases require a follow-up of at least two years to determine the final outcome of this surgical technique.

## CRediT authorship contribution statement

**Diego de Francisco Jiménez Cortes:** Writing – review & editing. **Edgar Manuel Bodu Lamberti:** Writing – original draft.

## Declaration of competing interest

None declared by the authors.
